# RCS reduction of wide-band, high-gain antenna array based on asymmetric transmission metasurfaces

**DOI:** 10.1038/s41598-024-64345-5

**Published:** 2024-06-11

**Authors:** Ting Wu, Lei Yan, Zikang Zhang

**Affiliations:** 1 Xi’an Key Laboratory of Wireless Optical Communication and Network Research School of Automation and Information Engineering, Xi’an University of Technology, Xi’an, Shaanxi China; 2 School of Automation and Information Engineering, Electronic Science and Technology Postdoctoral Research Center, Xi’an University of Technology, Xi’an, Shaanxi China

**Keywords:** Engineering, Optics and photonics

## Abstract

In this paper, an ultra-wideband stealth antenna with high gain on the basis of asymmetric transmission metasurface (ATMS) is proposed. ATMS can convert an incident *y*-polarised sphere wave into an *x*-polarised plane wave at the front side and controls the scattering of the incident y-polarised wave to the back side. Excitation of ATMS via a horn antenna, a low radar cross-section (RCS) and wideband antenna system is designed. Furthermore, through design of the meta-atoms and optimization of the macrosequencing, broadband RCS reduction is achieved. The experimental data indicated the reduction of the RCS of the antenna system by up to 10 dB and more than 20 dB in the frequency range of 10.1 GHz to 18 GHz (relative bandwidth is 56.2%) and 13.9 GHz to 18 GHz (relative bandwidth is 25.7%), respectively. In addition, a 3 dB gain relative bandwidth of 57.4% is achieved between 10 and 18 GHz, with a peak gain of 28.2 dB. It is noteworthy that the high gain and low scattering performance of the antenna are achieved in the same spectral range (10–18 GHz), and there is no interference between the scattering performance and radiation performance of the antenna, which could be controlled separately.

## Introduction

An antenna is an exceptional scatterer with the basic function of transmitting and receiving electromagnetic (EM) waves^1^. It is worth noting that antenna stealth cannot be sacrificed for radiation performance, so it is hard for only traditional scattering control techniques to effectively reduce the antenna's RCS^2–6^, For example, radar-absorbing materials, passive cancellation and shape stealth technology. Since emergence of metasurface (MS)^7–12^, they have demonstrated significant advantages in the antenna stealth domain in comparison to conventional scattering control technology due to their powerful EM wave manipulation capacity^13–15^. During the last years, MS offered new approaches towards cloaking realizations in the antenna field, making fruitful achievements in this area. By loading resistance onto the upper absorbing metasurface, a Fabry–Perot (FP) resonator antenna is engineered for realizing RCS reduction, the antenna's radiation performance will be significantly degraded^16^. A low RCS, polarization reconfigurable antenna that is based on a cross-slotted MS is proposed for improving the stealth performance of the antenna while limiting its bandwidth^17^. In Ref.^18^, the integration of a microstrip antenna and MS is proposed for conformal stealth antenna applications as a means of achieving low RCS and improving radiation performance. Although the radiation performance of the antenna is improved, the in-band stealth performance is unstable. Therefore, due to the contradiction between the scattering and radiating properties of the antenna, stealth antenna design faces great challenges, especially with wide-band properties.

An ultra-wideband stealth antenna with high gain on the basis of asymmetric transmission metasurface (ATMS) is proposed in this paper. First, through analyzing that the stealth antenna principle of operation, it was discovered that the backscatter beam and front should be independently adjusted. Therefore, an ATMS is needed for design and simulation. Second, the design method for the ATMS is researched. Converting patches through the component's polarization change, 2-bit transmissive coding elements are intended to adjust the front-incident wave to achieve beam focusing. In the meanwhile, through adapting the scatter control patches' lengths, achieve 180° phase difference between elements. Better backscattering reduction can be achieved by algorithm optimization. Third, an integrated ATMS with front beam focusing and backscattering reduction effects is designed. At last, this stealth antenna system was simulated, implemented, measured, also compared to other stealth antennas.

## Working principle of the stealth antenna

**Figure 1:**
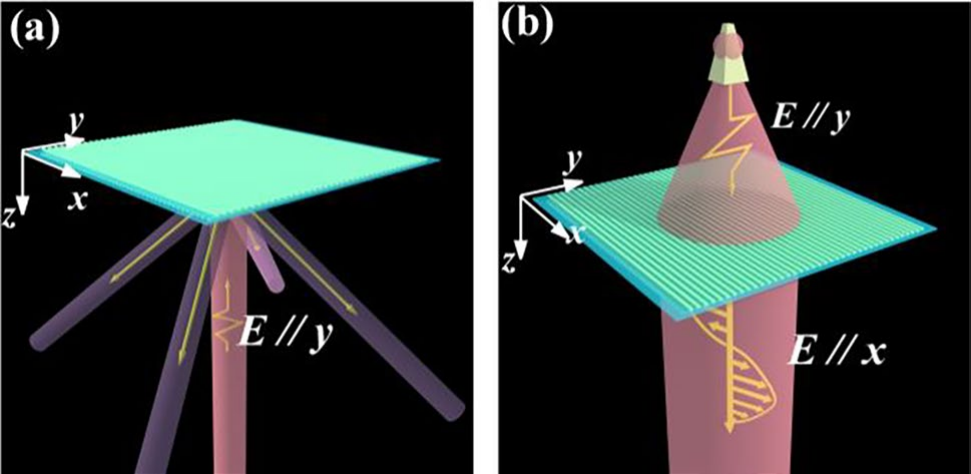
Schematics of the antenna based on ATMS. The antenna exhibits (**a**) stealth performance when excited by the bottom incident wave with *y*-polarization and (**b**) high-gain performance when excited by a *y*-polarized horn antenna.

The design of in-band stealth antennas faces great challenges due to the interference between the radiation performance and scattering characteristics^19^. Therefore, a new method is proposed for independently controlling antenna radiation and its scattering. Strong scattering occurs when the antenna system is excited by a *y*-polarized beam. Due to the strong beam control capability, an ATMS can be used for scattering the incident *y*-polarized beam. The *y*-polarized wave scatters in many directions when illuminating the back side of the ATMS, as shown in Fig. 1a. To control the scattering of the antenna without any deterioration in radiation performance, the method of polarization separation control is adopted, enabling the polarization of the antenna radiation wave and the scattering wave to cross-polarize with each other, as shown in Fig. 1a, b. This enables the radiation and scattering waves to be controlled independently.

It should be noted that when the stealth antenna radiates, the ATMS is equivalent to both a transmissive focusing MS and a transmission phase correcting device. It can convert the spherical wave that is excited by the feed at the focus into a plane wave. In addition, nonreciprocity is a characteristic of ATMS. When the same polarization incident wave excites the ATMS from different directions, the EM response exhibited by the ATMS is inconsistent. Under this condition, ATMS is designed to realize the aforementioned goal, which can be used to simultaneously control the transmission and reflection wave, and the scattering and radiation properties could be individually manipulated.

## Meta-atom design

**Figure 2:**
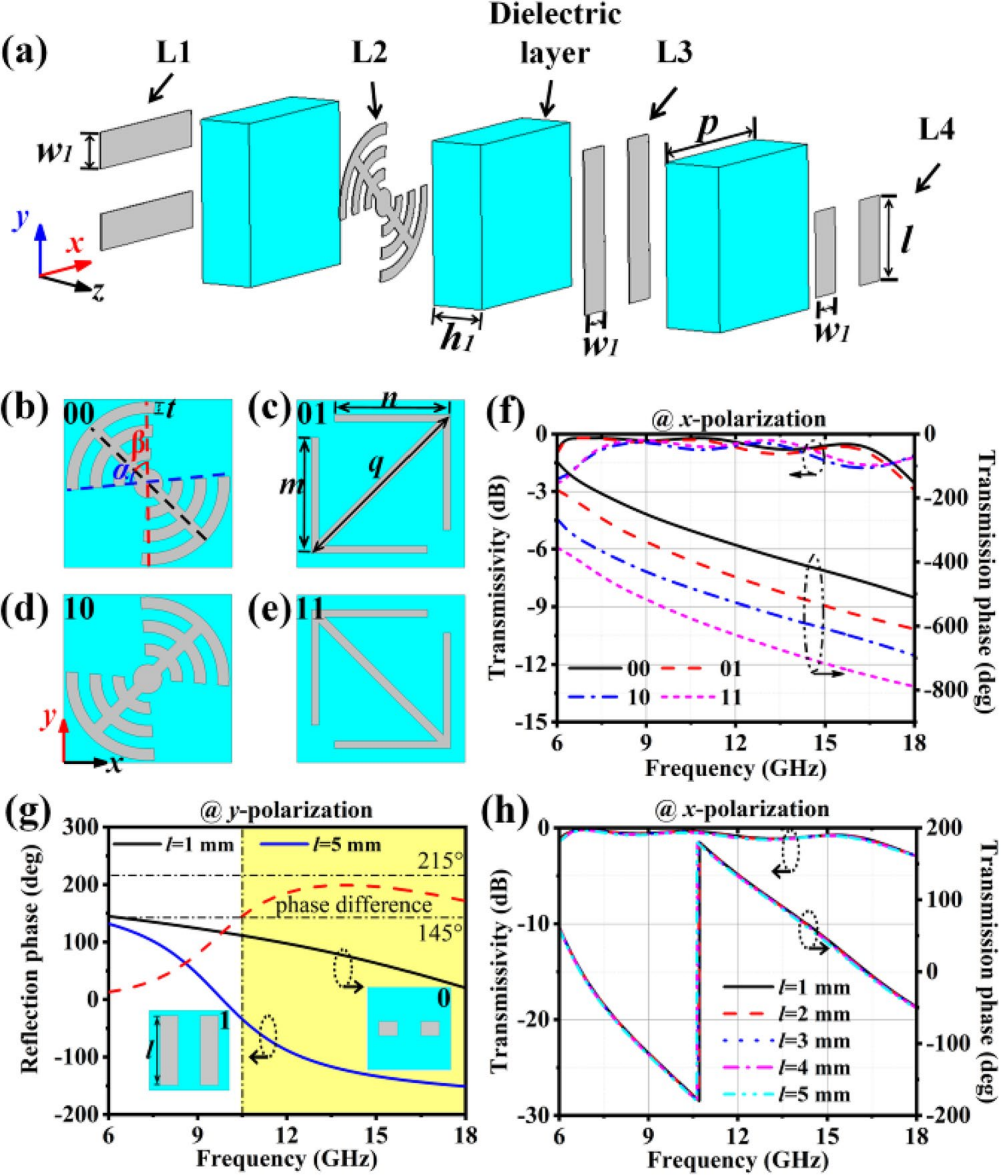
Structural parameters of the multi-layer meta-atom. The structural parameters of meta-atoms are: $\alpha = 45^\circ$, $\beta = 50^\circ$, $r = 2.8$ mm, $t = 0.4$ mm, $p = 5.8$ mm, $w_{1} = 1.3$ mm, $m = 4$ mm, $n = 4$ mm, $q = 6.5$ mm. (**a**) The overall structure of the meta-atom. (**b–e**) L2 layer metallic patch structure of four phase coding meta-atoms. (**f**) Cross-polarization (*x*-polarization) transmissivity and transmission phase curves of the 2-bit transmission phase coding meta-atoms. (**g**) the *y*-polarized reflection phase and phase difference of the 1-bit reflection phase coding meta-atoms and (**h**) effects of different *l* on cross-polarization transmission properties.

The fundamental aspect of the ATMS lies in meta-atom design. Inspired by our previous work^8^, a meta-atom simultaneously with the properties of high efficiency polarization conversion transmission and reflection manipulation which can independently control the transmitted as well as the scattered wave simultaneously is proposed as shown in Fig. 2a. First, the fundamental characteristics of the required meta atoms are analyzed according to the transmission mode shown in Fig. 1b. Second, the reflection mode modulation of the meta-atoms is gradually superimposed. Finally, the basic ATMS meta-atoms are obtained.

Firstly, the design features of meta-atomic efficient transmission and transmission polarisation conversion are investigated: Fig. 1b indicates that ATMS operates in polarization-switched transmission mode. And a metallic patch with bisymmetric characteristics would not exhibit any cross-polarization modes when exposed to y-polarised as well as x-polarised waves. While the patch is rotated by 45° or −45° respectively, the distinction is that the phase difference of the cross-polarized wave is 180° in both transmission and reflection modes^20^. The 1-bit phase modulation of the cross-polarized wave can be achieved when the patch is rotated. In addition, efficient cross-polarized transmission can be achieved by making adjustments to the distance between the metallic structures.

Second, the co-polarized reflection modulation characteristics of the meta-atom is analyzed. The metallic grating is completely transmissive to one polarization, while totally reflecting its orthogonal polarization. Therefore, a layer of raster-like metal structure is considered to stack above the metallic grating to realize reflection modulation of co-polarized wave.

Based on the above analysis, the meta-atom is finally optimized. The meta-atom is composed of a three-layer medium and four-layer metallic patches, as shown in Fig. 2a. The medium is an F4B plate ($$\varepsilon = 2.65$$$$\tan \delta = 0.001$$) with a thickness of $$h_{1} = 2\, mm$$. The metal is copper with a thickness of 0.036 mm. The metallic structure in the meta-atom is named the L1 layer, L2 layer, L3 layer and L4 layer. The L1, L2, and L3 layers form the transmitted polarization conversion function and focus function. In addition, transmitted trans-polarized waves enable 1-bit transmission phase modulation by rotating the polarization conversion layer by 90°. The L3 and L4 layers form the co-polarized reflection modulation function for the *y*-polarized wave. To achieve phase modulation for 2-bit transmission of the cross-polarized (*x*-polarization) wave, two types of L2 layer patches are designed, as shown in Fig. 2b–e.

In order to validate the proposed meta-atoms, simulations of the meta-atoms were carried out with assistance from the FDTD approach. Under illumination of the *y*-polarized wave, the transmittance shown in Fig. 2f obviously indicates that the *y*-polarized wave is transformed productively to a *x*-polarized wave and transmitted. The transmissivity is better than −1.5 dB in the frequency range of 8–16 GHz, which further indicates the capability of the meta-atoms for the efficient transmission of the *x*-polarized wave. Furthermore, as shown in Fig. 2f, the multilayer metallic structure, which is similar to the FP resonant cavity, generates multiple resonance points with small broadband fluctuations, and difference in phase among neighboring phase curves is 90°, which constitutes a basis of the realization of antenna broadband radiation. Figure 2g shows the *y*-polarized reflection phase of the meta-atoms. The meta-atom that contains the rectangular metallic patch is encoded with length “*l* = 1 mm” as “0”, and the meta-atom that contains the rectangular metallic patch is encoded with length “*l* = 5 mm” as “1”. It has been observed that among 10.5 GHz and 18 GHz, the phase difference across neighboring meta-atoms is 145° to 215°, Since the RCS reduction is at least 10 dB, it could be considered as a 1-bit situation in order to achieve the desired stealth effect^21^. As depicted in Fig. 2h, when the scattering control patch length *l* is changed, the phase and transmittance of the cross-polarisation transmission are never deteriorated significantly. This indicates that the meta-atoms modulate the phase of the reflected and transmitted wave simultaneously. It should be noted that, this scattering control layer installed on the front side of the metallic grating layer parallel to the *y*-axis can be regarded as a broken grating structure, which has no modulation effect on the *x*-polarized wave. The simulation results show that these meta-atoms can be used to design high-efficiency broadband stealth antenna.

## Ultra-wideband stealth antenna design

### A. Antenna radiation and scattering control array design

On the basis of the above meta-atom design, the stealth antenna of an ultra-wideband has been devised. The array consists of 45 × 45 elements with an overall dimension of 261 × 261 mm^2^, and a horn antenna in the frequency range of 8–18 GHz has also been employed as the feed source.

**Figure 3:**
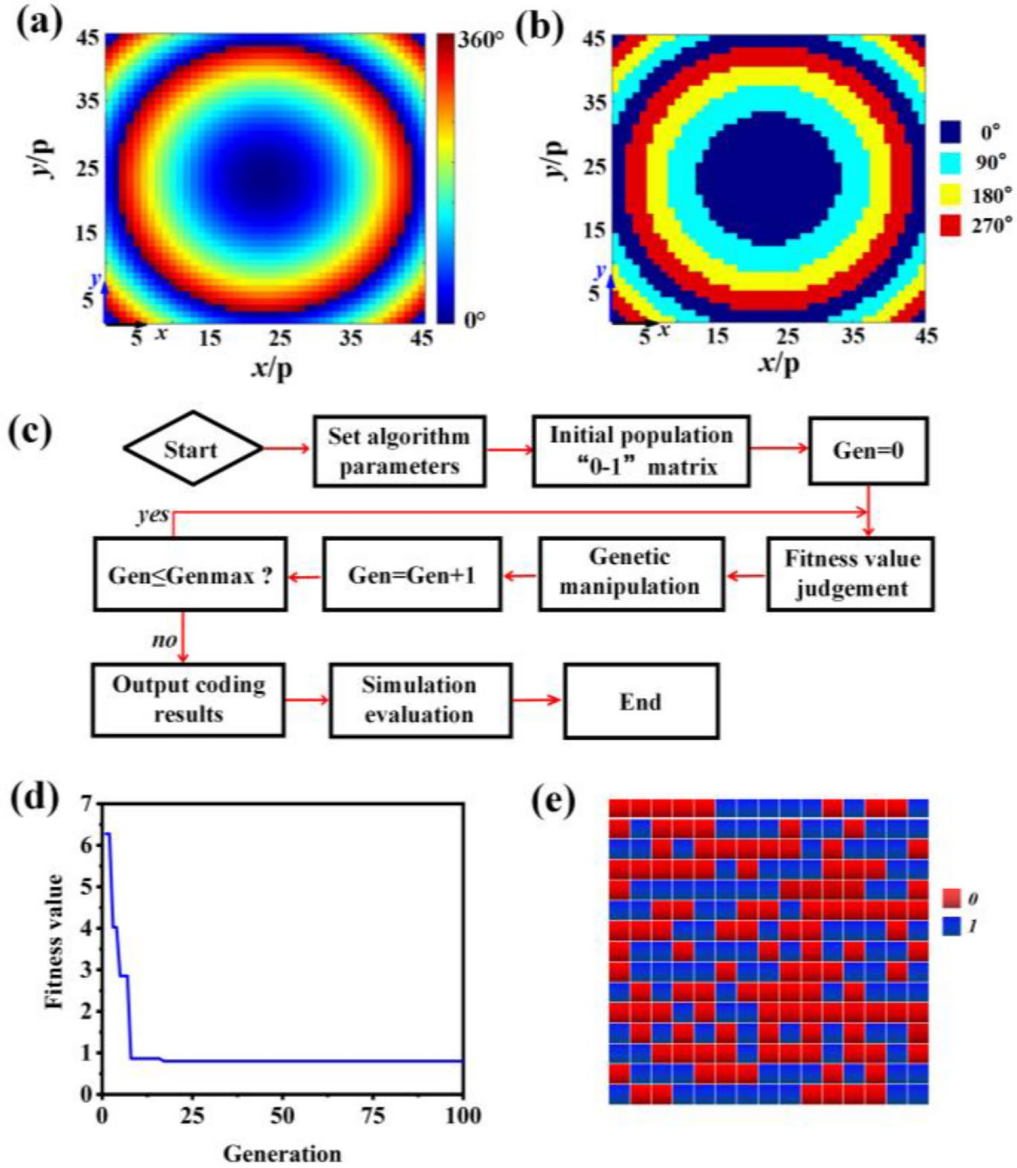
(**a**) The transmission phase distribution at 14 GHz of antenna array. (**b**) The discrete 2-bit coding phase distribution of antenna array. (**c**) The algorithm optimization flowchart. (**d**) The relationship between the fitness value and number of iterations. (**e**) Optimized 1-bit phase coding of scattering control layer. “0” indicates the phase is 0° and “1” indicates the phase is 180°.

Firstly, this stealth antenna radiation array is designed. The antenna's transmission phase distribution can be obtained from Eq. (1) and shown in Fig. 3a.

1 $$\phi (m,n) = \frac{2\pi }{\lambda } \cdot (\sqrt {(m \cdot p)^{2} + (n \cdot p)^{2} + F^{2} } - F).$$

$\lambda$ represents the free-space wavelength for the operating frequency, *n* and *m* represents the number of meta atoms in the *y-* as well as *x-*directions etc., *p* represents the unit period, while *F* = 280 mm represents the focus position in Eq. (1). After that, 2-bit discrete phase encoding is performed using 90° as the 2-bit discrete interval. The final radiation array arrangement is presented in Fig. 3b. It should be noted that the center frequency is 14 GHz. The distance between the horn’s phase center and the horn’s aperture plane is 75 mm. Therefore, the distance between the horn’s aperture plane and antenna array is 205 mm. The antenna radiation array is constructed with the centre of the feed phase placed at the focal point.

Then, the effective controlling of antenna scattering was investigated. An optimization algorithm was applied to optimize the 1-bit reflection phase encoding, as shown in the flowchart for the algorithm in Fig. 3c. The back-scattering field function for the antenna array is expressed as Eq. (2).

Except for backscattering, the mean function of the scattering field in other directions of the antenna array is expressed as Eq. (3). The fitness function (cost function) is expressed as Eq. (4). The incident wave direction is oriented perpendicular to the antenna array. Each parameter in Eqs. (2), (3) and (4) is described as follows: $f_{e} (\theta_{i} ,\phi_{i} )$ stands for the incident field function, $\varphi (m,n)$ represents the reflection phase of every meta atom, and $(\theta_{bs} ,\phi_{bs} )$ represents backscattering direction under custom coordinates. In the calculation, the step depth can be adjusted to 1° using Eq. (4) with $\theta \in (0,90^\circ )$ and $\phi \in (0,360^\circ )$. $S_{n}$ stands for the count for angular coordinates.

Following the optimisation of the algorithm, this dependence among the number of iterations and the fitness value, as illustrated in Fig. 3d. When the fitness function value is stable and no longer changes, the optimized scattering control patch arrangement is output, as shown in Fig. 3e. At last, an optimised 1-bit phase-encoded scattering control array was devised.

2 $$f_{bs} (\theta_{bs} ,\phi_{bs} ) = f_{e} (\theta_{i} ,\phi_{i} )\sum\limits_{m = 1}^{N} {\sum\limits_{n = 1}^{N} {e^{{ - i\left\{ {\varphi (m,n) + kd\sin \theta_{bs} \left[ {(m - \frac{1}{2})\cos \phi_{bs} + (n - \frac{1}{2})\sin \phi_{bs} } \right]} \right\}}} } {\kern 1pt} }$$

3 $$f_{av} (\theta ,\phi ) = \frac{{\sum\limits_{{\theta \ne \theta_{bs} }} {\sum\limits_{{\phi \ne \phi_{bs} }} {\left\{ {f_{e} (\theta_{i} ,\phi_{i} )\sum\limits_{m = 1}^{N} {\sum\limits_{n = 1}^{N} {e^{{ - i\left\{ {\varphi (m,n) + kd\sin \theta \left[ {(m - \frac{1}{2})\cos \phi + (n - \frac{1}{2})\sin \phi } \right]} \right\}}} } {\kern 1pt} } } \right\}} } }}{{S_{n} }}$$

4 $$fitness{\kern 1pt} {\kern 1pt} {\kern 1pt} {\kern 1pt} {\kern 1pt} {\kern 1pt} = k_{1} \cdot f_{bs} (\theta_{bs} ,\phi_{bs} ) + k_{2} \cdot f_{av} (\theta ,\phi )$$

### B. Antenna radiation performance results

**Figure 4:**
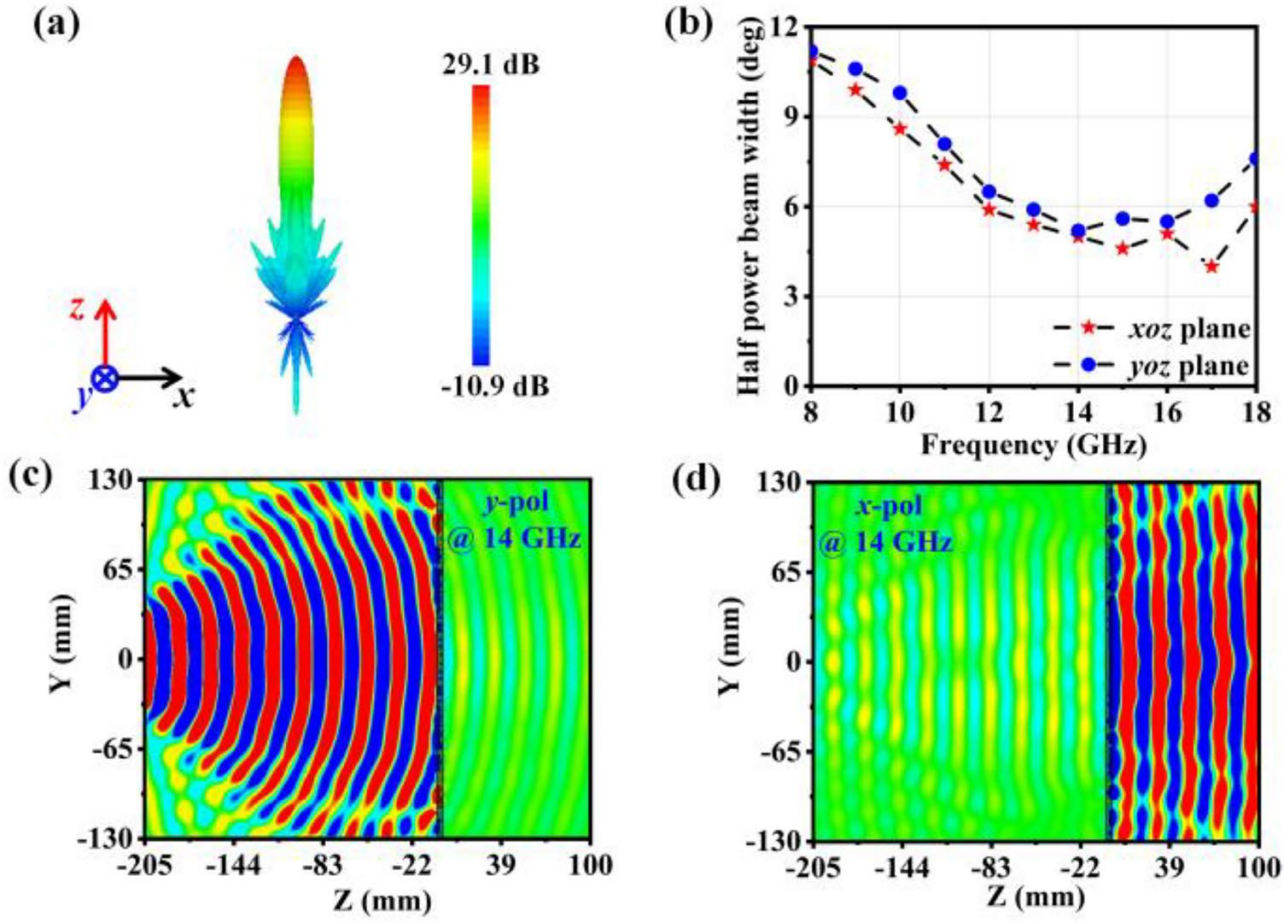
Simulation results of antenna radiation performance. (**a**) Feeding source radiating a y-polarised wave propagating in the + z direction with a three-dimensional far-field radiation pattern at 14 GHz. (**b**) Half-power beam width (*xoz* and *yoz* plane) of the antenna in 8–18 GHz. (**c,d**) Antenna arrays convert spherical waves into plane waves at 14 GHz, as shown by the electric field.

Upon installation, a scattering control array, radiation array as well as feed are mounted together to constitute an antenna system. In order to verify the antenna's radiation performance, digital simulations were conducted by using the FDTD method. The far-field radiation performance at 14 GHz is illustrated in Fig. 4a, in which the antenna can radiate a fine pen-beam with a peak gain of 29.1 dB. The half-power beamwidths in the *xoz* and *yoz* plane are displayed in Fig. 4b. Figure 4c, d are schematic diagrams of Re(Ex) and Re(Ey) in the *yoz* plane. The antenna-emitted Y-polarised spherical wave is transformed into an X-polarised plane wave as it passes over the array, agreeing with previous analyses. All these above findings indicate that this proposed antenna shows efficient radiation performance of high broadband gain.

### C. Antenna scattering performance results

**Figure 5:**
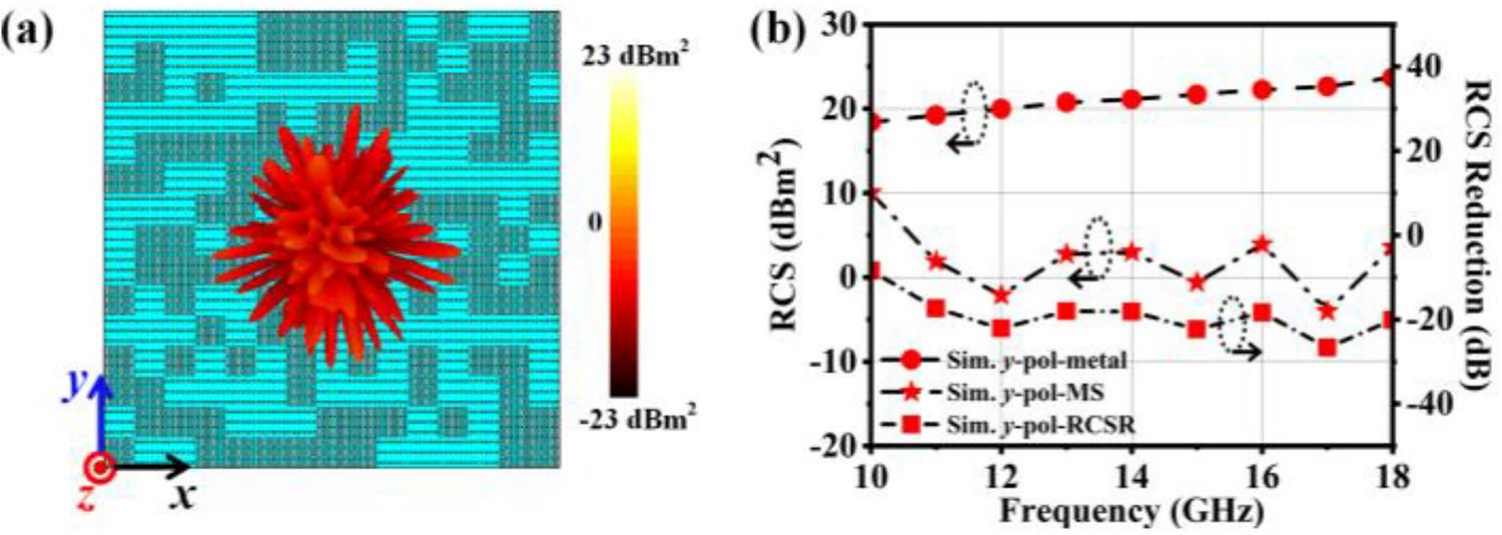
Simulation results for the radiation performance of the antenna. (**a**) When y polarised waves propagate through -*z* direction, the three-dimensional far-field scattering pattern at 14 GHz. (**b**) RCS of the antenna and a metal plate of the same size, as well as the RCS reduction of the antenna compared with the same-sized metallic plate from 10 to 18 GHz range as a y-polarised wave propagates in the -*z* direction.

In order to verify the properties of the antenna for diffuse scattering, a *y*-polarized wave is set propagating along the -*z* direction to irradiate the antenna array, which is shown in Fig. 5a in the scattering simulation results. The 3 × 3 basic elements are set to the same reflection phase to form a meta-atom, and then the 15 × 15 meta-atoms are arranged according to the distribution shown in Fig. 5a. As shown by far-field scattering pattern at 14 GHz in Fig. 5a, the diffuse scattering effect of the antenna is obvious. The illuminated *y*-polarized plane wave is randomly scattered in every direction. Therefore, it can effectively decrease antenna's backward RCS. To quantify the characteristics of the RCS of the antenna, metal plates of the same size is placed to the same electromagnetic conditions in order to compare and verify. Some difference between the backward RCS of the antenna and a same-sized metallic plate is used to evaluate the backscattering intensity of the antenna. Then the RCS reduced value is shown in Fig. 5b. The RCS reduction value reaches above10 dB from 10 to 18 GHz (relative bandwidth is 57.4%), reaches more than 20 dB at 12 GHz, 15 GHz, 17 GHz and 18 GHz, and reaches 26.7 dB at 17 GHz. The proposed antenna exhibits outstanding properties of low RCS in Ku-band and X-band for Y-polarised waves.

The above results prove that the designed antenna has excellent RCS reduction characteristics for a *y*-polarized wave. It should be noted that when the antenna array is illuminated by the *y*-polarized wave, the reason for RCS reduction is that the scattering control layer can manipulate the reflection wave and realize the reflection-type RCS reduction.

## Fabrication and measurement

**Figure 6:**
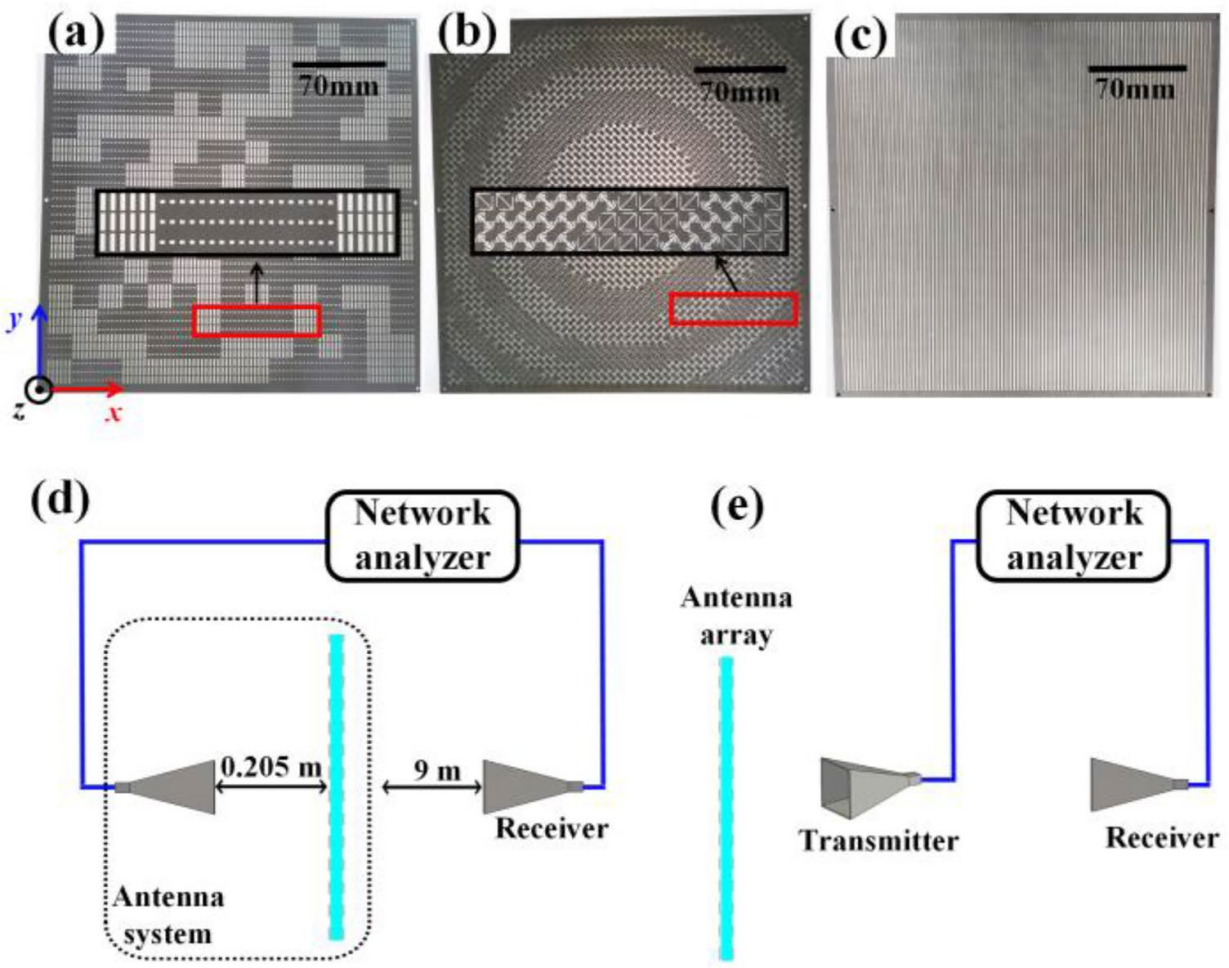
The antenna sample and the theoretical experimental diagram. (**a**) Scattering control layer. (**b**) Polarization conversion layer. (**c**) Metallic grating layer. Measurement diagrammatic sketch of the (**d**) radiation far-field and (**e**) scattering far-field.

In order to provide further verification on the performance of the proposed antenna system, a prototype was physically implemented and experimentally measured. The components of the sample are shown in Fig. 6a–c. The four-layer structures, starting from the bottom to the top, the L1, L2, L3 and L4 layers. The four-layer metal fabrication is interconnected with F4B ($\varepsilon = 2.65$, $\tan \delta = 0.001$). The prototype experimental system is shown in Fig. 6d, e. To minimize the influence of clutter from the surroundings, the prototypes produced was measured within anechoic chamber.

**Figure 7:**
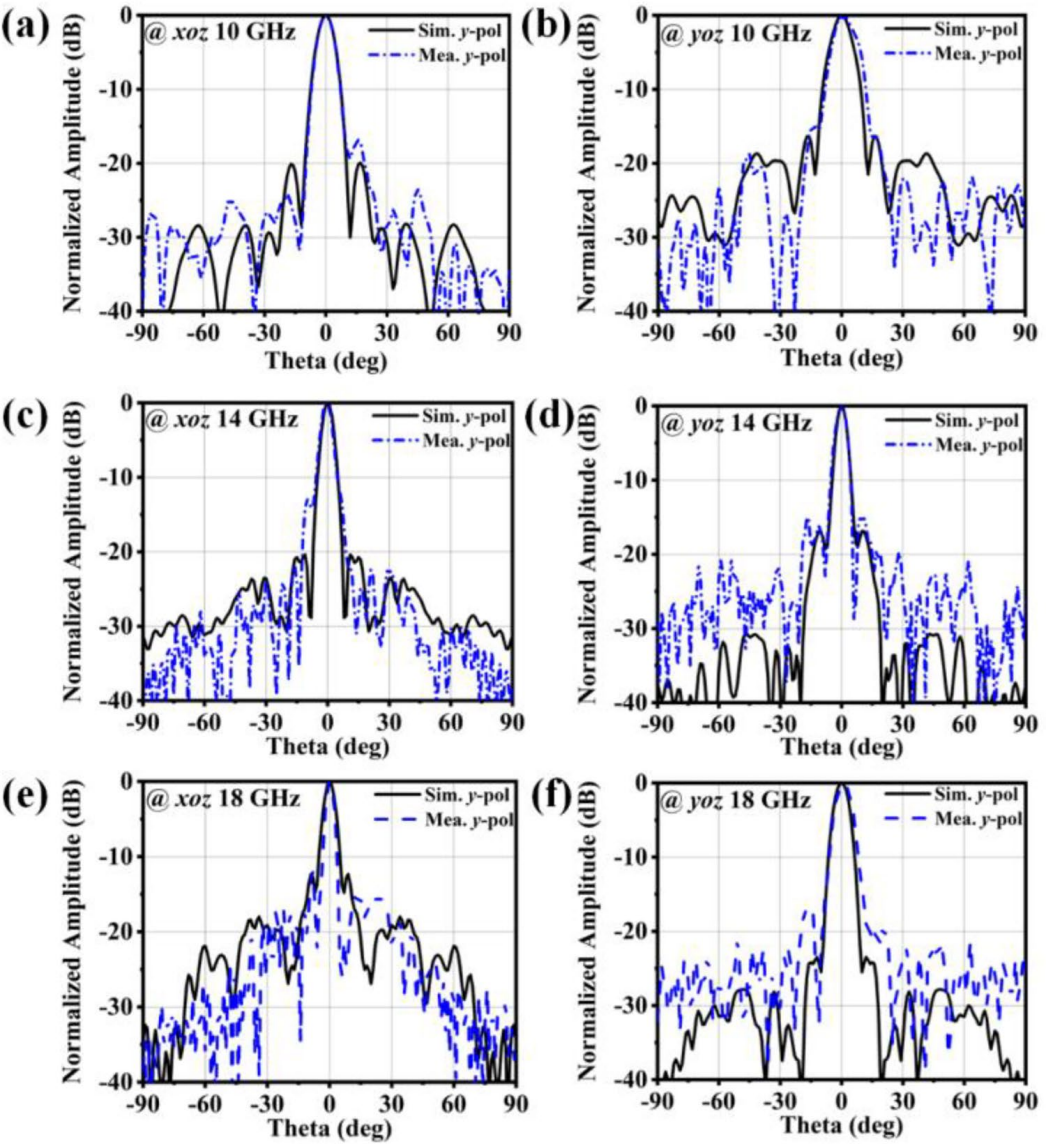
The measured and simulated results for the radiation performance of the antenna. Radiation far-field pattern of (**a**) *xoz* and (**b**) *yoz* plane at 10 GHz. Radiation far-field pattern of (**c**) *xoz* and (**d**) *yoz* plane at 14 GHz. Radiation far-field pattern of (**e**) *xoz* and (**f**) *yoz* plane at 18 GHz.

**Figure 8:**
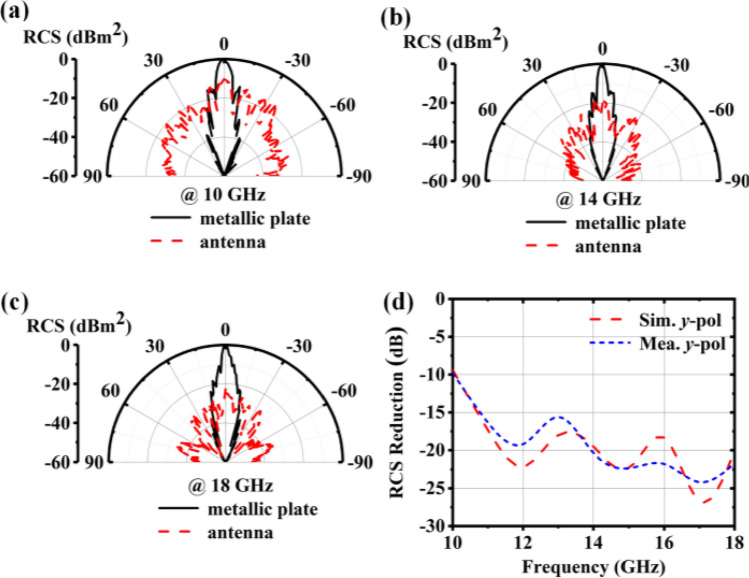
The measured and simulated results for the radiation performance of the antenna. (**a**) Realized gain and (**b**) polarization isolation obtained by simulation and measurement.

An experimental study of the antenna system's scattering and radiating properties was carried out. First, the radiation performance was characterized. As shown in the schematic diagram of the radiation performance measurement in Fig. 6d, the feed antenna is placed 205 mm away from the array, and the *y*-polarized wave is launched from the feed. The receiver is a linear polarization double ridge horn antenna that operates at 1–18 GHz. As depicted in Fig. 7a–f, the simulated and measured far-field patterns in the *xoz* and *yoz* planes at 10 GHz, 14 GHz and 18 GHz demonstrate exquisite high gain radiation results and stability of the fine radiation performance of the antenna. At the center frequency of 14 GHz, the antenna’s gain is 10 dB higher than that of the horn antenna feed. Apparently, these results adequately validate that the antenna is available for achieving high-gain broadband radiation. As depicted in Fig. 8a, the results of the simulated and measured realized gain show a measured 3 dB gain relative to bandwidth of 57.4% with a peak gain of 28.4 dB. The results reveal excellent ultra-wideband high-gain stability in the Ku-band and X-band. As depicted in Fig. 8b, the measurement polarization isolation is relatively stable at approximately 25 dB, which verifies the low cross-polarization level in the entire Ku-band and X-band and proves that the antenna can achieve high-polarization-purity wideband radiation. Because of the polarization isolation characteristics, the metallic grating used by the meta-atoms can improve the polarization purity of the radiation wave.

**Figure 9:**
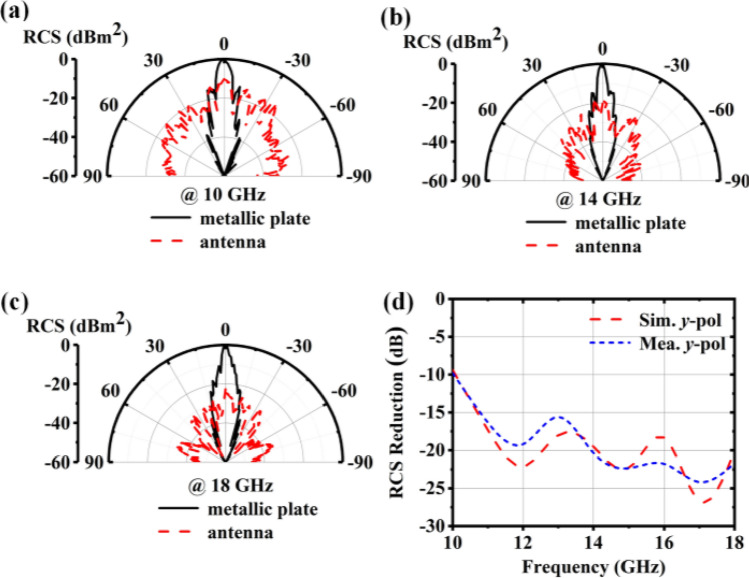
The measured results of antenna scattering performance. (**a–c**) Scattering far-field pattern of *xoz* plane at 10 GHz, 14 GHz and 18 GHz obtained by measurement. (**d**) The antenna RCS reduction when *y-*polarized wave propagates along the -*z* direction illuminating the antenna array.

Second, the scattering performance was characterized. As shown in Fig. 6e, to avoid affecting the effect of backward RCS measurement, the transmitter deflects from the normal direction of the antenna array at a fixed angle of approximately 5°^8^. Figures 9a–c illustrate the two-dimensional scattering far-field patterns at 10, 14 and 18 GHz. To summarize, the antenna achieves a low backward RCS at Y-polarization, and it’s scattering performance exhibits excellent stealth characteristics. measured results indicate that this antenna's backward RCS reaches 20.3 dB at a centre frequency of 14 GHz. In addition, the backward RCS is reduced by 9.8 dB at 10 GHz, while it is reached by 22.3 dB at 18 GHz. Moreover, the backward RCS reduction reaches a peak value of 24.3 dB at 17.2 GHz. As shown in Fig. 9d, a 10 dB RCS reduction bandwidth is validated on a metal plate of the same size. This obviously indicates that the backward RCS reduction exceeds 10 dB in the frequency range of 10.1–18 GHz (relative bandwidth is 56.2%) and more than 20 dB in the frequency range of 13.9–18 GHz (relative bandwidth is 25.7%).

Finally, the stealth antenna system is compared to other stealth antennas, as shown in Table 1. From this table, the improved performance of the stealth antenna compared with other reported works can be concluded as follows:

**Table 1:** Comparison with other stealth (low RCS) antennas.

Ref. no.	Antenna form	Polarization	Aperture size (at center frequency)	Peak gain (dB)	3 dB gain bandwidth (%)	Peak in-band RCS reduction (dB)	10 dB RCS reduction bandwidth (%)
^22^	MA	Linear	3.5*λ* × 3.5*λ* (10 GHz)	16.7	–	–	–
^23^	MA	Linear	7.5*λ* × 7.5*λ* (15 GHz)	23.1	29.3	25	48.6
^24^	MA	Linear	4.2*λ* × 4.2*λ* (5.7 GHz)	9.4	–	21.2	32
^25^	MA	Circular	0.6*λ* × 0.6*λ* (6 GHz)	7.5	< 10	22.96	< 10
^26^	MA	Circular	1.3*λ* × 1.3*λ* (5.2 GHz)	–	23.9	30.9	< 10
^9^	MA	Linear	2.4*λ* × 2.4*λ* (6 GHz)	9.8	–	18.2	< 10
^27^	MA	Linear	4*λ* × 4*λ* (5 GHz)	22	< 10	7	–
^7^	MA	Linear	3.7*λ* × 3.7*λ* (10 GHz)	19.8	4.8	16.4	< 10
Our work	MA	Linear	13*λ* × 13*λ* (14 GHz)	28.2	57.4	24.3	56.2

*MA* metasurface antenna.

1. The 3 dB gain bandwidth of the antenna and the 10 dB RCS reduction bandwidth lie in the same spectral range (10–18 GHz), and the relative bandwidths both reach more than 55%.
2. The improvement in the antenna stealth performance does not lead to deterioration of antenna radiation performance. It solves the contradiction between scattering as well as radiation performance in all process of antenna design.

## Conclusion

Consequently, an ATMS-based in-band stealth antenna is designed to operate within the ultra-broadband range. The proposed antenna system considers both the scattering and radiation properties, low in-band RCS and high-efficiency radiation are achieved within the ultra-wideband. In the military field, especially in the application of reconnaissance and antireconnaissance, the stealth performance of communication equipment is often the cornerstone of a surprise victory. Moreover, such an antenna system is applicable to multiband communication systems that operate within the Ku-band and X-band. The approach described in this paper could apply to the design of stealth antennas and other meta devices in other spectrums as well.
